# The investigation of effect on foot plantar massage on functional recovery in older adults with general surgery, randomized clinical trial

**DOI:** 10.1007/s40520-024-02770-2

**Published:** 2024-05-23

**Authors:** Asuman Saltan, Selda Mert, Önder Topbaş, Beyza Aksu

**Affiliations:** 1 Department of Physiotherapy and Rehabilitation, Faculty of Health Sciences, Yalova University, Yalova, Turkey; 2 Department of Nursing, Faculty of Health Sciences, Kırşehir Ahi Evran University, Kırşehir, Turkey; 3 Department of Medical Services and Techniques, Kocaeli Vocational School of Health Services, Kocaeli University, Kocaeli, Turkey; 4 Vocational School of Health Services, Kocaeli University Hospital, Kocaeli University, Kocaeli, Turkey

**Keywords:** Aging, Massage, Kinesiophobia, Nurses, Rehabilitation

## Abstract

**Objective:**

Foot massage is known to be effective on the emotional state (anxiety, depression, etc.) in the postoperative period. However, studies on its effect on functional level are insufficient.

**Aim:**

The study aimed to investigate the impact of foot plantar massage on functional recovery in older adults undergoing general surgery, employing a randomized clinical trial design.

**Methods:**

A total of 70 older adults aged 65 years and above who underwent abdominal surgery were included. Various assessments were conducted, including pain levels (Visual Analogue Scale), fear of mobility (Tampa Scale for Kinesiophobia), functional independence (Functional Independence Measure), balance (Berg Balance Scale), basic mobility (Rivermead Mobility Index), mental function status (Standardized Mini-Mental State Examination), and delirium (Nu-DESC).

**Results:**

Statistically significant differences were observed in some assessment parameters within the groups during the 2nd and 3rd measurement times, with the intervention group demonstrating significant mean differences.

**Discussions:**

The literature underscores the increase in kinesiophobia scores post-general/abdominal surgery in older adults, emphasizing the importance of evaluating functional level and kinesiophobia to expedite discharge processes and potentially plan early post-discharge rehabilitation to mitigate readmissions for functional reasons.

**Conclusions:**

Ultimately, foot massage was found to be effective in reducing kinesiophobia, improving balance, mobility, daily living skills, and mental status in older adults post-abdominal surgery, thereby advocating for the facilitation of post-discharge rehabilitation programs or the reduction of readmission rates.

**The clinical trials number:**

NCT05534490.

## Introduction

The elderly stage represents the ultimate phase of human existence, a natural and unalterable journey encompassing physiological, psychological, and socio-economic dimensions [1]. By the year 2023, one out of every ten individuals in Turkey will be aged 65 or above, with the 85-plus demographic being the most rapidly expanding segment of the population [2, 3]

One of the important factors of healthy ageing is considered to be the ideal functional level of the individual [4]. The functional level of older people is influenced by a wide range of factors, from chronic disease to socio-economic status. The independence of the older people’s daily life; not being dependent on someone to provide personal care and the organisation of the older people home or living environment is associated with healthy aging [5]. However, emergencies brought about by declining physiology may affect functionality. Surgical interventions are an approach that suddenly moves the regular life in the opposite direction [4, 6]. One of the factors affecting the functional level of the individual is surgical procedures [4, 6]. A review of the literature has shown that loss of function occurs not only in older people after lower extremity surgery, but also after cardiac, cancer and abdominal surgery [4–7]. According to Tan et al., the functional level lost after surgery in the older people may become permanent even if the operation site heals. In this case, evaluation of the functional level in the older people after surgery and making appropriate decisions may prevent both early discharge and re-hospitalisation [4].

Functional level assessments in the older people are frequently included in the literature [4, 6, 7]. These assessments include in addition to the commonly used daily life assessments and balance and psycho-mental assessments. Moreover, it is known that these evaluations are performed after certain surgical procedures, especially after surgical procedures involving the lower extremities [4, 6, 7]. However, it has been reported in the literature that functional evaluations should also be performed after surgeries involving the heart, cancer, and abdominal region [4, 8]. It is therefore important for to identify these factors before is prescribed any post-operative rehabilitation order or before a patient is readmitted to hospital [4, 8].

The importance of functional evaluations after surgery in older people is also important for finding treatment methods that may be effective on the functional level. There are studies in the literature that suggest that massage therapy can be effective at a functional level in older people [9–11]. Massage therapy could improve mobility, allowing patients to perform daily activities, making rehabilitation easier [9, 10]. The utilization of massage therapy has seen a notable rise as a method to facilitate the mobilization of anatomical structures such as muscles and subcutaneous tissues, achieved through the application of mechanical force [12]. This therapy elicits venous return and lymphatic movement, diminishes swelling, and promotes the mobilization of skin, tendons, and muscle fibers. Consequently, its effects encompass pain reduction, stress and anxiety alleviation, and the induction of muscle relaxation, which has demonstrated improvements in sleep quality and the pace of functional recovery. Nevertheless, research on the effects of foot massage on functional performance in older individuals remains sparse to date [12, 13]. A lack of clear evidence regarding the effectiveness of foot massage exists, thereby preventing the recommendation of this regimen in the postoperative care of patients who have undergone Abdominal Surgery. To elucidate the potential benefits of foot massage in clinical settings, a randomized clinical trial was conducted with the aim of assessing whether patients undergoing Abdominal Surgery experienced improved functional recovery upon receiving foot massage. Valuable insights from our findings could inform clinical strategies for managing patients who have undergone Abdominal Surgery.

## Method

### Design and participants

The Ethics Committee of Kocaeli University granted approval for this study and all patients provided their consent. We evaluated patients who underwent AS at Kocaeli University Hospital between October 2021 and May 2022 for eligibility. The inclusion criteria consisted of individuals meeting the following conditions: (1) age exceeding 18 years, (2) undergoing post-surgery care for a duration exceeding 1 day, and (3) having undergone abdominal surgery. Subjects with a history of diabetes exceeding 10 years or those afflicted with cerebrovascular, neurological, or psychiatric disorders were excluded due to the perceived substantial adverse effects of long-term diabetes on surgical outcomes [14]. Only eligible participants, individuals aged 65 years or older who are able to comply with study procedures and assessments, have no hearing or visual impairments, and have voluntarily agreed to take part, were recruited for this study.

### Data collection

The determination of sample size was guided by prior research [15], with a confidence coefficient of 95%, a confidence interval of 1.96%, and a Type II margin of error set at 10% (working power of 90%). Consequently, the calculated sample size was 32 individuals per group, with an allowance of 3 potential dropouts per group. Upon obtaining approval from participants through the informed consent form, indicating their agreement with “I agree,” the study commenced on a voluntary basis.

Block randomization was carried out by an impartial research scientist.

The intervention group and the control group each comprised an equal number of patients, with 35 individuals allocated to both groups through random assignment. Patients in the intervention group received massage treatment until discharge, which was based primarily on the patients’ length of stay in hospital. Typically, our hospital discharges patients who undergo abdominal surgery four days after the operation.

### Intervention

On the second day post-surgery, the intervention group was directed to assume a supine position and undergo a five-minute whole-body relaxation procedure [9, 10, 16]. Subsequently, each foot was massaged with sweet almond oil, a commonly used lubricant in massage therapy, for a duration of 5–10 min. A pillow was utilized to elevate the patients’ feet during the massage. The massage technique encompassed both the plantar and dorsal aspects of the foot. Initially, the therapist positioned the fingers on the dorsal aspect while placing the thumbs on the plantar aspect. Using gentle pressure, one thumb stroked upward on the plantar region of the foot. Beginning from the heel and progressing to the toes, with the sole of the foot facing the therapist, the therapist’s fingers were positioned on top of the toes, repeating the procedure 5–10 times per session [9, 10, 16]. The massage was administered by an experienced therapist to ensure uniformity among all patients. Routine care was provided to both groups, with no additional massage administered to other body regions. The massage sessions were conducted on a daily basis.

Data were collected regarding the demographic characteristics, pain levels, kinesiophobia, functional performance, balance, mobility, mental status and delirium levels of individuals both before surgery and intervention, as well as on the day of discharge (three measurements were obtained). Data regarding the type and duration of anaesthesia administered, as well as information about the patient’s age and Body Mass Index (BMI), were acquired from the patients’ files.

Individuals completed measurements to evaluate pain, kinesiophobia, functional performance, balance, mobility, mental level and delirium before and after the intervention. Pain levels were assessed using a Visual Analogue Scale (VAS), fear of mobility was assessed using the Tampa Scale for Kinesiophobia (TSK), functional independence in daily activities was evaluated using the Functional Independence Measure (FIM), balance was evaluated by Berg Balance Scale (BBS), basic mobility in daily life was assessed using the Rivermead Mobility Index (RMI), mental function status was evaluated using the Standardized Mini-Mental State Examination (SMMSE), delirium was evaluated by via Nu-DESC. Abbreviations were explained upon first use. Assessments were conducted through face-to-face interviews carried out by a qualified a physiotherapist and nurse.

The Visual Analogue Scale (VAS) was utilized to gauge individuals’ subjective evaluation of their pain levels. Various previous studies have employed it to assess pain severity and have established its reliability and validity. Aslan et al., first investigated the sensitivity and selectivity of VAS following general surgery in Türkiye and determined a Cronbach alpha value of 0.88 [17].

Participants were instructed that a score of “0” on the scale denotes the absence of pain, while a score of “10” indicates the most severe pain imaginable. The degree of pain corresponds with the numerical value assigned. Subsequently, the participants were asked to indicate their level of pain intensity using a number from 0 to 10 [17, 18].

The assessment of kinesiophobia was conducted using the Turkish version of the Tampa Scale of Kinesiophobia (TSK), a 17-item questionnaire. Respondents were prompted to indicate their level of agreement with each of the 17 statements on a four-point response scale, ranging from ‘strongly disagree’ to ‘strongly agree.’ Notably, four items (4, 8, 12, and 16) featured reverse-worded statements. The TSK, a validated instrument for measuring fear of movement, yields scores ranging from 17 to 68. A high level of kinesiophobia is indicated by scores above 37, according to recent studies. The TSK has undergone rigorous testing on chronic pain patients, demonstrating strong internal consistency, temporal stability, as well as face and content validity [19, 20].

The Functional Independence Measure (FIM) analyses two aspects of impairment, motor and mental function, to assess functional independence in daily activities. The FIM comprises six functional sections: self-care, sphincter control, mobility, locomotion, communication, and social perception. Functional independence is assessed across eighteen activities using a seven-point scale, with a maximum achievable score of 126. Elevated scores indicate increased independence in daily life activities. Widely employed in medical rehabilitation, the FIM serves as a prominent activity assessment tool [21].

The Berg Balance Scale (BBS), a widely utilized clinical assessment tool, was originally devised to evaluate balance in older adults [22]. Comprising 14 items, the test entails the performance of static and dynamic everyday tasks of varying difficulty levels. Each item is rated on a scale of 0 to 4, with a maximum total score of 56 denoting superior balance [23].

The RMI is a metric that assesses mobility. It objectively evaluates all the variables associated with movement, ranging from simple actions such as turning in bed to more complex ones like running. This assessment includes 14 questions and one observation, all organized in a hierarchical manner. Each affirmative answer earns one point, and scores can range from 0 to 15. Objective and value-neutral language is used throughout. Technical term abbreviations are explained when first used, and style guide rules are followed. A score of 15 indicates zero mobility issues, while scores of 14 or below indicates the presence of one. Research has demonstrated the soundness and dependability of the RMI, as well as its practicability among older adults. The present study revealed a Cronbach alpha value of 0.91 [24].

The SMMSE offers a comprehensive evaluation of mental capacity. The assessment is easily implementable and provides insight into the extent of cognitive damage. The test has a maximum score of 30, and higher numbers indicate a better mental status. Scores below 23/24 indicate abnormal cognition in the Turkish population. The Revised SMMSE is administered to older adults with less than five years of education, while the Regulated SMMSE is administered to educated individuals. A score of 24–30 is classified as normal cognitive function, a score of 20–23 as mild cognitive impairment, and a score less than 19 as moderate cognitive dysfunction [25].

The Nu-DESC consists of five items that focus on the nurse’s observations of the patient and assess disorientation, inappropriate behaviour - communication, illusions/hallucinations, and psychomotor slowing. A score between 0 and 2 is assigned for each item, with a maximum score of 10 [26]. The Nu-DESC form, used in many countries, is solely based on person observation and takes approximately 1 min to complete. Moreover, training is not necessary for utilising this scale. The Turkish validation and reliability assessment of Nu-DESC was performed in 2019 by Çınar and Eti Aslan, establishing a sensitivity of 92.3% and a specificity of 92.7% [27].

### Statistical analysis

Parametric data were expressed as the mean ± standard deviation (SD) and were subjected to comparison utilizing suitable statistical tests, such as Student’s t-test or Wilcoxon signed rank test. Non-parametric data were represented by the mean, with p-values obtained from the Chi-square test or Fisher’s exact test. Effect size was determined employing Phi, calculated by taking the square root of the chi-squared statistic divided by the sample size. A threshold for clinical relevance was established at an effect size of ≥ 0.4.

## Results

The study enrolled 70 subjects, randomised 35 to the intervention group and 35 to the control group. Analysis of Table 1 showed no significant differences in baseline characteristics such as sex, age, body mass index (BMI), percentage of type and duration of anaesthesia, and length of hospital stay between the two groups. The average age was 64.2 ± 4.1 years for the intervention group and 64.8 ± 3.8 years for the control group. The intervention group exhibited a gender distribution of 31.4% female (*n* = 11) and 68.6% male (*n* = 24), while the control group displayed a gender distribution of 42.9% female (*n* = 15) and 57.1% male (*n* = 20).

**Table 1:** Demographic characteristics of the patients analyzed

Variable		Study group		*p* value
		Intervention group	Control group (*n* = 35)	
Age (years)		66.2 ± 2.8	66.5 ± 2.2	0.941
Gender, n (%)	Female	11 (31.4%)	15 (42.9%)	1.00
	Male	24 (68,6)	20 (57.1)	
BMI		26.9 ± 3.8	28.0 ± 4.4	0.458
Devices of Walking	Yes	2 (5.7%) (Walking stick)	2 (5.7%) (Walking Stick)	0.328
	No	33 (94.3)	33 (94.3)	
Number of devices of walking		11 (32.4%)	16 (42.1%)	0.468
Time of discharge		88.5 ± 62.5	113.8 ± 80.5	0.147
Time of anhestesis		73.8 ± 19.6	113.8 ± 50.5.	0,018

* *p* < 0.05, BMI: body mass index

There was no statistically significant difference found in the comparison of measurement times pre-operative (1st measurement) to post-operative (2nd measurement), and discharge periods (3rd measurement)) between the groups in pain assesment (*p* = 0.293, *p* = 0.190, *p* = 0.898). Statistical analysis indicates a substantial distinction (*p* = 0.000) within each group during the second and third measurement periods, even though both groups demonstrated comparable outcomes.

A statistically significant difference was observed between the groups in the evaluation of kinesiophobia when comparing the measurement times. Specifically, the values measured at the 1st and 2nd measurement times exhibited a significant difference (*p* = 0.020 and *p* = 0.018, respectively), whereas no significant difference was found between the groups at the 3rd measurement time (*p* = 0.673).

Statistically significant differences were found when evaluating the 2nd and 3rd measurement times within the groups. Specifically, the intervention group demonstrated a significant difference (*P* = 0.000), while the control group did not show any significant difference (*p* = 1.000). These results are displayed in Table 2.

**Table 2:** Comparison of VAS, TSK, FIM, RMI, SMMSE, BBS, Nu-DESC score averages in Intervention and Control groups (*n* = 70)

		Intervention Group	Control Group	*p* value
VAS	Pre-operation	6.77 ± 1.53	6.22 ± 1.88	0.293
	Baseline	2.54 ± 1.55	2.48 ± 2.11	0.190
	Post-intervention	2.88 ± 2.55*	3.34 ± 1.23*	0.898
	p value	0.000	0.000	
TSK	Pre-operation	42.80 ± 5.69	39.11 ± 7.18	0.020
	Baseline	41.40 ± 5.42	42.05 ± 7.40	0.673
	Post-intervention	38.08 ± 5.95*	41.97 ± 7.34*	0.018
	p value	0.000	0.000	
FIM	Pre-operation	114.42 ± 21.64	113.48 ± 10.99	0.356
	Baseline	102 ± 26.49	86.05 ± 25.15	0.729
	Post-intervention	113.22 ± 20.28*	93.11 ± 22.76*	0.025
	p value	0.000	0.000	
BBS	Pre-operation	39.48 ± 8.27	37.17 ± 6.96	0.210
	Baseline	33.91 ± 16.34	27 ± 16.55	0.083
	Post-intervention	38.57 ± 12.21*	27 ± 16.52	0.001
	p value	0,000	1,000	
RMI	Pre-operation	13.68 ± 1.47	13.17 ± 1.70	0.550
	Baseline	11 ± 3.93	8.77 ± 4.83	0.910
	Post-intervention	13 ± 1.92*	8.97 ± 4.74	0.000
	p value	0.000	0.090	
SMMSE	Pre-operation	24.88 ± 3.70	23.54 ± 4.50	0.168
	Baseline	23.22 ± 4.28	20.94 ± 4.66	0.808
	Post-intervention	24.94 ± 3.69*	21.20 ± 4.73	0.256
	p value	0.000	0.080	
Nu-DESC	Pre-operation	0.17 ± 0.38	0.25 ± 0.44	0.084
	Baseline	0.57 ± 0.50	0.42 ± 0.50	0.390
	Post-intervention	0.17 ± 0.38*	0.22 ± 0.42*	0.238
	p value	0.000	0.019	

* *p* < 0.05, Visual Analogue Scale (VAS), Tampa Scale for Kinesiophobia (TSK), Functional Independence Measure (FIM), Berg Balance Scale (BBS), Rivermead Mobility Index (RMI), Standardized Mini-Mental State Examination (SMMSE), Nursing Delirium Screening Scale (Nu-DESC).

There was no statistically significant difference found between the groups when the values measured at the 1st and 2nd measurement times were compared in the evaluation of functional independence (*p* = 0.356 and *p* = 0.729, respectively). However, a statistically significant difference was observed between the groups at the 3rd measurement time (*p* = 0.025). When the second and third measurement times were assessed within the groups, a statistically significant difference was found in both groups (*P* = 0.000) (Table 2).

No statistically significant difference was found in the balance evaluation when comparing the measurement times between the groups for the values measured at the 1st and 2nd measurement times (*p* = 0.210, *p* = 0.083 respectively). However, a statistically significant difference was observed between the groups for the 3rd measurement time (*p* = 0.001). When evaluating the 3rd and 3rd measurement times, a statistically significant difference was observed in the intervention group (*P* = 0.000), while no significant difference was found in the control group (*p* = 1.000) as shown in Table 2.

There was no statistically significant difference found between the groups in mobility evaluation when the measurement times were compared for values measured at the 1st and 2nd measurement times (*p* = 0.550, *p* = 0.910, respectively). However, at the 3rd measurement time, a statistically significant difference between the groups was observed (*p* = 0.000). Statistically significant differences were observed in the intervention group when evaluating 2nd and 3rd measurement times within the group (*P* = 0.000), while no significant difference was found in the control group (*p* = 0.090) (Table 2).

There were no significant differences found among the values measured at all measurement times when comparing mental levels between the groups (*p* = 0.168; 0.808; 0.256). However, evaluating the second and third measurement times within the groups, there was a statistically significant difference found in the intervention group (*P* = 0.000), but not in the control group (*p* = 0.080) (Table 2).

No statistically significant differences were found between the groups at any point during the evaluation for delirium (respectively, *p* = 0.084, *p* = 0.390, *p* = 238). A statistically significant difference was found in both groups (*P* = 0.000; 0.019) when the 2nd and 3rd measurement times were evaluated within groups (Table 2).

## Discussion

In this study, kinesiophobia and functional level after general surgery in older people were evaluated and the effectiveness of foot massage on these factors was assessed. As a result of the evaluations, it was determined that foot massage application was effective on kinesiophobia, balance, mobility level, daily living skills and mental level in the postoperative period involving the abdominal region in older adults. Wijeysundera et al., in their study of functional assessment after surgery in 2022, stated that a screening method should be developed to quickly identify older people at risk of functional decline after surgery [28]. However, it is understood that the author conducted the functional assessments in his study through the use of individual statements rather than the scale. Sayılan et al., suggested that the evaluation of functional levels among older people after surgery should be standardised and that there is a need for studies that include an analysis of whether the values of the functional parameters in question can be changed by the applications to be made [7]. In this research, massage was administered on the plantar surface of the foot following functional assessments. Our results indicate the effectiveness of massage on the functionality of older people. No analogous studies were discovered in the literature.

Pain has been identified in the literature as a contributing factor to kinesiophobia [29]. The ability to move decreases in some individuals due to pain. This situation is particularly common after surgery and in older people. In the study carried out by Koraş and Karabulut in 2018, examining the impact of foot massage on abdominal surgery pain, statistical analysis showed no significant differences in mean pain values, which were 6.18 in the experimental group and 6.28 in the control group on the first day, as measured with VAS [30]. A comparison of pre-operative pain scores in the Turkish population showed results that were similar to our study. In the present study, the pain level was recorded as 6.77 for the experimental group and 6.22 for the control group, and no significant difference was found between the two groups. Although the mean values of VAS after the application were slightly higher than the values after the surgery when the mean values of VAS were compared between the groups, the fact that there was a significant difference between the 1st and 3rd measurements and there was no difference between the 2nd and 3rd measurements shows that massage may not have an effect on pain (Table 2). Systematic reviews and meta-analyses in the literature also document this condition [8, 12]. There was no significant statistical difference between the groups when analysing the mean pain scores in this study, either before or after the intervention. It was found to reduce pain in both groups. Thus, it is arguable that the effect of foot massage on pain in older people after abdominal/general surgery remains a topic of debate. The existing literature on the use of massage therapy for the treatment of pain in individuals suggests that it may promote relaxation and reduce anxiety. There is a lack of standard protocols, short intervention periods of one to five days or hours, limited outcome measures and neglected outcomes [12, 30–33].

The importance of pain in this study is revealed when the kinesiophobia values are analysed. Although pain decreased in both groups, fear of movement was consistently higher than the literature’s average value for both groups during all measurement periods (> 35 TSK) [19]. However, a statistically significant reduction between pre- and post-intervention scores was observed in the intervention group when TSK scores were analysed (Table 2). The pain decreased over time in both groups, but the levels of kinesiophobia were still high. There is no treatment for kinesiophobia after general surgery in the literature. However, studies on kinesiophobia caused by pain and musculoskeletal problems are also limited. In this way, our study prepares the ground for the literatüre (35). However It has been highlighted in the literature that kinesiophobia scores increase after general/abdominal surgery in the older people [7] especially the importance of assessing the functional level and kinesiophobia in the older people in order to speed up the discharge process and, possibly, to plan early for the rehabilitation period after discharge and to reduce the return to hospital for functional reasons [3, 7, 32, 33]. This study is significant as the only one to suggest that foot massage may be effective in the treatment of kinesiophobia in the post-operative period in the older people. According to Xu et al. [34]., multi-modal therapies may be more effective for kinesiophobia. Based on this, the literature suggests that massage therapy may have an effect on the emotional state of the individual, such as anxiety and depression [3, 7, 32–34]. This study has shown that massage is also effective on functional factors. We believe that massage therapy after general surgery in elderly people could be considered as an effective method both psychologically and physically.

The level of function is one of the most important factors that should be assessed during the hospitalisation of older people, regardless of the reason for their hospitalisation [28]. Knowledge of an individual’s level of functioning is a determining factor in the length of their stay in hospital, the potential for readmission after discharge, and even the risk of mortality [28, 35]. Therefore, it is very important to determine the functional levels of the older people in the hospital and to make interventions accordingly [28]. In this study, participants presented with good preoperative functional levels. Subsequently, both groups experienced postoperative FIM values declining below 100. However, FIM values increased to 100 during the discharge period for the intervention group, as compared to the control group where levels stayed below 100. Significantly, this disparity held statistically. It can be concluded that the use of foot massage is effective in improving functional level after abdominal/general surgery in the older people [13, 15]. The underlying mechanism can be explained as follows. It has been reported in the literature that the effects of foot massage on the older people are effective in electroencephalography evaluations, which are the brain signals of the individual [36], stimulating the pressure receptors under the feet, affecting static balance and postural control, thus providing psychological confidence and physiological stimulus for the individual to move [13, 15].

Foot massage for older people has been shown to improve postural control and balance [37, 38]. The literature includes balance as a factor linked with kinesiophobia and the risk of falls in older people [38–40]. In 2017, Miller et al. reported that older people experienced a loss of balance and mobility scores, particularly in the first week after gynaecological surgery [41]. In the analysis conducted in this study, the balance parameter showed higher values in the intervention group compared to the control group during the discharge period. Based on this, it can be concluded that massaging the plantar surface of the feet can be effective in older people [36, 41]. It has been suggested that tactile stimulation of the plantar surface of the foot has an effect on body perception and postural control, and may therefore have an effect on static and dynamic balance [38]. Thus, individuals’ motivation to move could be supported by reducing their kinesiophobia [38].

There was also a correlation between pain in the ankle or knee joints and the functionality of the individuals. In this scenario, an intervention targeting the lower extremities, such as the foot and ankle, is likely to improve functionality, assuming that these specific body parts are experiencing functional impairments [39, 43–46]. The study discovered the potential effectiveness of foot massage in improving balance.

In 2017, however, Miller et al., reported that there is a risk of falls in people following gynaecological surgery [41]. Similarly, studies conducted by Lawrence et al. (2004) and Staggs, Mion, and Shorr (2015) suggest that surgery can result in functional limitations for individuals [47, 48]. However, previous studies have indicated a requirement for examining all parameters of functional assessment, such as measurements of pain and functional limitations [40, 47, 48]. In this study, we have laid the foundation for future research by assessing balance-mobility and kinesiophobia through functional dependences.

The evaluation was based on questionnaires designed to provide numerical values for pain, kinesiophobia, mobility, and other factors. However, these values do not have clinically significant meaning. For example, the study by Parker et al. introduced the concept of the minimal clinically important difference (MCID) to gauge the essential threshold for attaining treatment efficacy [49, 50]. Definitive conclusions on the effect of foot massage intervention on clinical outcomes cannot be made as we did not associate them. To address this, the use of MCID may be necessary. Additionally, the study has limitations such as a relatively short follow-up period. Therefore, further investigation is required to evaluate the long-term effects of foot massage in postoperative patients. Furthermore, future studies should also assess whether foot massage can benefit other complications, such as sleep disorders. Despite its limitations, this study is the first in its field and will serve as a foundation for future research.

## Conclusion

As a result of the evaluations, it was determined that foot massage application was effective on kinesiophobia, balance, mobility level, daily living skills and mental level in the postoperative period involving the abdominal region in older adults. This aims to ease and expedite the drawing up of rehabilitation programmes after discharge or to lower readmission rates [28, 50].

## Data Availability

No datasets were generated or analysed during the current study.
